# Variability in Inferior Vena Cava (IVC) Filter Retrieval Rates and Adherence to Guidelines: A Multi-Center Retrospective Analysis

**DOI:** 10.3390/jcm14124034

**Published:** 2025-06-07

**Authors:** Zachary Nuffer, Phil Ramis, Eric M. Rohren, Gary Horn

**Affiliations:** 1 Department of Radiology, Baylor College of Medicine, Baylor St. Luke’s Medical Center, 1101 Bates Ave., Houston, TX 77030, USA; 2 Radiology Partners Research Institute, 2330 Utah Avenue Suite 200, El Segundo, CA 90245, USA; phil.ramis@radpartners.com

**Keywords:** inferior vena cava filter retrieval, guideline adherence, interventional radiology

## Abstract

**Background/Objectives**: Inferior vena cava (IVC) filters are used to prevent pulmonary embolism (PE) in patients with contraindications to anticoagulation, among other indications. Despite clinical guidelines recommending timely retrieval, rates remain suboptimal, raising concerns about long-term complications. This study examines national hospital-level variation in IVC filter retrieval rates and adherence to timing recommendations. **Methods**: A retrospective analysis was conducted using data from 12,197 patients across 158 U.S. facilities between 1 January 2020, and 29 August 2024. Retrieval rates, time to retrieval, and associations with facility-specific factors such as procedural volume and academic affiliation were evaluated using descriptive statistics and correlation analyses. **Results**: Facility retrieval rates varied widely, ranging from 0.36% to 100%, with a mean of 23% (SD 24%). Only 43% (SD 12%) of filters were retrieved within 90 days, as recommended. A weak negative correlation was observed between retrieval rate and procedure volume (r = −0.24), as well as patient age (r = −0.17). Several high-volume facilities showed potential guideline non-adherence, placing many filters but retrieving few. No significant differences were found between academic and non-academic institutions in retrieval rate or timing. **Conclusions**: Substantial variability exists in IVC filter retrieval practices, and many facilities fall short of timely removal benchmarks. These findings highlight the need for targeted quality improvement initiatives to increase retrieval adherence, reduce filter-related complications, and improve patient outcomes.

## 1. Introduction

Inferior vena cava (IVC) filters are widely utilized as a prophylactic intervention to prevent pulmonary embolism (PE) in patients who have contraindications to anticoagulation therapy, among other indications. Initially introduced as permanent implants, the evolution of filter technology has led to the development of retrievable devices intended for short-term use during periods of transient thromboembolic risk.

The failure to retrieve IVC filters when clinically appropriate can result in significant long-term morbidity. Retained filters are associated with a spectrum of serious complications, including device fracture, filter embolization, IVC thrombosis, chronic venous insufficiency, and penetration of the caval wall into adjacent structures such as the aorta, duodenum, or vertebral body [1]. These adverse events can necessitate complex surgical or endovascular retrieval procedures, increase healthcare utilization, and cause permanent harm to patients. Importantly, many of these complications are preventable through timely filter removal.

In response to accumulating evidence of these adverse events, the U.S. Food and Drug Administration (FDA) issued a safety communication in 2010 and updated guidance in 2014, recommending prompt removal of retrievable filters once protection from PE is no longer necessary—ideally within 29 to 54 days of placement [2]. Multiple professional societies have echoed these recommendations, emphasizing the importance of timely filter retrieval as a best practice standard. The Society of Interventional Radiology (SIR), in its 2020 Clinical Practice Guideline for IVC Filters, emphasizes the importance of timely retrieval, stating that retrievable filters should be removed “as soon as clinically appropriate” and within a timeframe that balances the risk of recurrent thromboembolism with potential device-related complications [3]. While the guideline does not prescribe a rigid interval, it supports removal within a 29–54-day window in most cases and recognizes that retrieval within 90 days is a reasonable upper threshold in routine clinical practice. Similarly, the American College of Chest Physicians (ACCP), in their 2016 evidence-based guidelines on antithrombotic therapy for venous thromboembolism, recommends that temporary IVC filters be retrieved once anticoagulation is feasible or the risk of PE has diminished. Although the ACCP guidelines do not specify an exact timeframe, they reinforce the principle that filters should not be left in place longer than necessary due to risks associated with long-term implantation [4].

Despite these clear recommendations, national adherence to retrieval guidelines remains inconsistent. Retrieval rates vary widely across institutions, ranging from <5% to over 50%, depending on institutional policies, presence of dedicated filter tracking systems, and provider engagement [5,6,7].

Numerous factors have been implicated in this variability, including differences in hospital infrastructure, academic affiliation, procedural volumes, patient demographics, and socioeconomic barriers [8]. Understanding these patterns is critical for informing quality improvement initiatives and reducing preventable long-term morbidity associated with IVC filter retention.

This study aims to characterize real-world retrieval practices across a diverse sample of U.S. healthcare settings. Specifically, we analyze retrieval rates, timing in relation to guideline-recommended windows, and associated institutional and patient-level factors. By identifying systemic and modifiable barriers to filter retrieval, this study contributes actionable insights toward optimizing the safe and effective use of IVC filters nationwide.

## 2. Materials and Methods

### 2.1. Study Design and Data Source

This was a multi-center retrospective analysis of IVC filter placements and retrievals across facilities in the United States from 1 January 2020 to 29 August 2024. Data were extracted from a Radiology Partners database containing patient demographics, filter placement and retrieval dates, and facility characteristics (location, academic affiliation, procedural volume). Clinical information such as patient comorbidities, functional status, or contraindication to filter retrieval were not available.

### 2.2. Inclusion and Exclusion

Patients were included if they had a filter placed during the study interval. This included patients who had a filter placed and subsequently died before retrieval or who had retrieval performed at a non-affiliated facility. Patients with incomplete demographic or facility data were excluded. Facilities were categorized as academic or non-academic based on their medical training program affiliation. In total, 158 facilities and 12,197 patients were included.

### 2.3. Study Outcomes

Retrieval rate: percentage of placed filters that were retrieved.

Adherence to 90-day retrieval guidelines: filters retrieved within 90 days.

Retrieval interval: mean number of days from placement to retrieval.

Academic vs. non-academic: comparison of retrieval rates and adherence.

Correlation of retrieval rates with procedure volume.

Correlation of retrieval rates with patient age.

### 2.4. Statistical Analysis

Descriptive statistics were used to summarize facility-level performance. Correlation analyses assessed relationships between retrieval success and patient age, retrieval interval, and academic status. T-tests and chi-square tests compared retrieval performance between academic and non-academic institutions.

### 2.5. Declaration of Generative AI

During the preparation of this work the authors used ChatGPT 4.0 by OpenAI in order to perform background research, analyze the data, and create the figures. After using this tool, the authors reviewed and edited the content as needed, and take full responsibility for the content of the publication.

## 3. Results

### 3.1. Retrieval Rate and Timing

Among 158 facilities and 12,197 patients, facility-level retrieval rates ranged from 0.36% to 100%. The mean retrieval rate was 23.2% (SD 24.3%). The median retrieval interval was 74 days (IQR: 41–121). Of the retrieved filters, 13.6% were removed within 30 days, 29.0% between 31 and 90 days, 24.6% between 91 and 180 days, and 31.2% after 180 days. When stratified by age, retrieval rates were highest among patients aged <50 years (3/3, 100%) and ≥80 years (1/2, 50%), though these groups had very small sample sizes. Among more populous age groups, retrieval rates were 272 out of 1469 (18.5%) for patients aged 50–59, 1688 out of 9760 (17.3%) for those aged 60–69, and 118 out of 962 (12.3%) for those aged 70–79, demonstrating a decreasing trend in retrieval rate with increasing age.

### 3.2. Facility Comparisons

No significant difference in retrieval rates or intervals was observed between academic and non-academic sites (*p* > 0.05). A weak negative correlation existed between retrieval rate and procedural volume (r = −0.24, *p* = 0.0024).

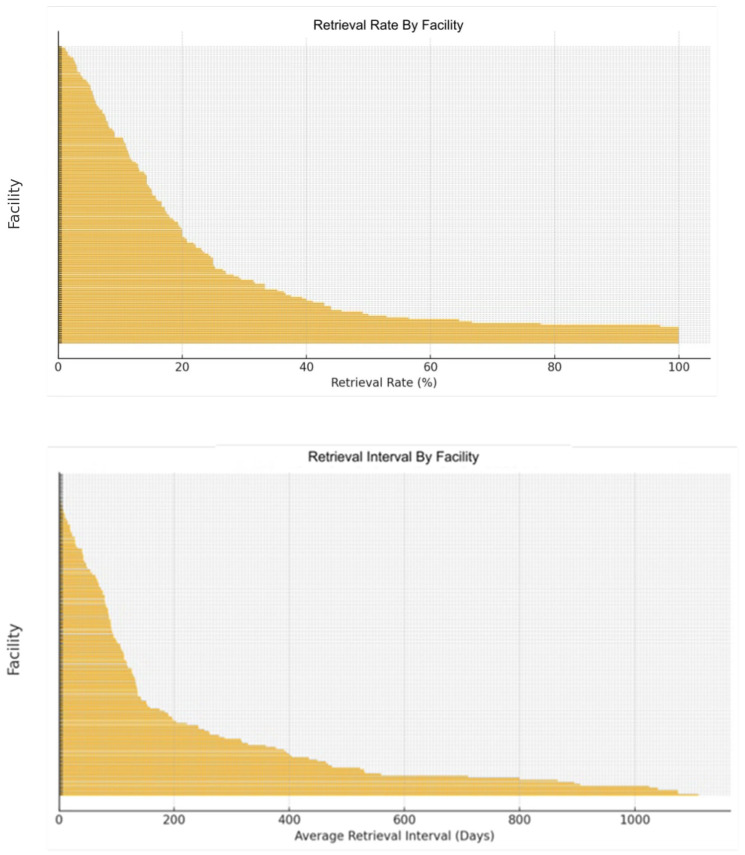



## 4. Discussion

The findings of this study align with the existing literature, underscoring the persistent challenges in IVC filter retrieval and adherence to clinical guidelines. Despite recommendations for timely retrieval, retrieval rates remain low and long-term complications associated with prolonged filter retention continue to be a concern. For this reason, the Society of Interventional Radiology guidelines recommend the use of a structured follow-up program to increase retrieval rates and detect complications. Studies have shown that these programs significantly improve retrieval rates, often exceeding 60–80% compared to national averages below 35% [8,9,10]. They also promote standardized decision making, better communication among providers, and improved patient awareness.

Our study identified a mean retrieval rate of 23% (standard deviation [SD], 24%), which is consistent with previous reports. A systematic review from 2012 reported an average retrieval rate of 34%, indicating that many IVC filters remain in place longer than recommended [8]. Similarly, a national cohort study observed a one-year cumulative retrieval incidence of 18.4%, further highlighting the gap between recommended and actual retrieval practices [7]. Our findings are consistent with those of previous studies, suggesting that little progress has been made over the past decade, and a substantial proportion of filters remain unretrieved, exposing patients to the risks associated with residual filters. 

The risks associated with prolonged IVC filter retention are well-documented. Studies have reported that up to 20% of patients experience filter-related complications, including deep vein thrombosis (DVT), filter migration, and vena cava perforation [9]. New DVT events have been reported to occur in up to 30% of patients and IVC thrombosis rates range from 6 to 30% [11,12]. Furthermore, as filter endothelialization and embedment progress, retrieval becomes increasingly challenging, with success rates diminishing significantly beyond 90 days post placement [13].

Our analysis also revealed significant variability in retrieval practices across facilities, with retrieval rates ranging from 0.36% to 100%. The weak negative correlation observed between retrieval rate and the total number of placement procedures performed at a facility suggests that as placement volume increases, retrieval may not scale proportionally unless specific systems are in place to manage follow-up, especially in populations that are fragmented or mobile (e.g., being treated at a tertiary center) or clinically complex. Older patient populations exhibited a weak negative correlation with retrieval rates (r = −0.17), suggesting that age may be a minor but relevant factor influencing the likelihood of retrieval. This observation is corroborated by national data showing that increasing age and certain comorbidities contribute to lower retrieval rates [7]. Additionally, geographic and institutional variations have been documented in prior studies, indicating that facility-specific protocols and regional practices play a role in retrieval success [8].

A notable weakness of our analysis is the possible inclusion of patients who had a filter placed and subsequently died before retrieval could be attempted, although we suspect that this number is small given that the overall 90-day mortality rate in a broad population with venous thromboembolism (VTE) and filter placement from the PREPIC 2 trial was 12.1% [14]. In addition, while large and multi-center in nature, our study uses only data from facilities affiliated with Radiology Partners, which may not be fully representative of national trends. For this reason, we were also unable to track patients who may have had their filters retrieved at outside institutions, which could result in underestimation of retrieval rates. Additionally, important clinical variables—such as indication for filter placement, comorbidities, and anticoagulation status—were not available, precluding multivariate analysis.

The findings of this study highlight a persistent gap between the clinical guidelines and real-world practice. The U.S. Food and Drug Administration (FDA) recommends removing retrievable IVC filters once the risk of pulmonary embolism subsides to prevent long-term complications [2]. Furthermore, the implementation of the Medicare Access and CHIP Reauthorization Act (MACRA) in 2015 and its Merit-based Incentive Payment System (MIPS) in 2017 were intended to improve healthcare quality through performance-based reimbursement and include a specific measure requiring documentation of plans for IVC filter removal at the time of placement. Despite these recommendations, our data demonstrated that retrieval rates remain low, indicating the continued need for improved follow-up protocols and patient management strategies. 

## 5. Conclusions

Significant variability in IVC filter retrieval practices persists, with many facilities failing to meet guideline-recommended retrieval timelines. The low retrieval rates observed in our study are consistent with published rates from prior studies and underscore the continued need for targeted interventions, such as structured follow-up protocols and patient education, to enhance retrieval adherence and minimize complications associated with prolonged IVC filter retention.

## Data Availability

The data supporting this study are available upon request from the corresponding author.

## Abbreviations

The following abbreviations are used in this manuscript:

IVC	Inferior vena cava filter
DVT	Deep vein thrombosis
PE	Pulmonary embolism

## References

[1] Angel L.F., Tapson V., Galgon R.E., Restrepo M.I., Kaufman J. (2011). Systematic review of the use of retrievable inferior vena cava filters. J. Vasc. Interv. Radiol..

[2] U.S. Food and Drug Administration (FDA) (2024). Removing Retrievable Inferior Vena Cava Filters: FDA Safety Communication. https://www.fda.gov/.

[3] Kaufman J.A., Barnes G.D., Chaer R.A., Cuschieri J., Eberhardt R.T., Johnson M.S., Kuo W.T., Murin S., Patel S., Rajasekhar A. (2020). Society of Interventional Radiology Clinical Practice Guideline for Inferior Vena Cava Filters. J. Vasc. Interv. Radiol..

[4] Kearon C., Akl E.A., Ornelas J., Blaivas A., Jimenez D., Bounameaux H., Huisman M., King C.S., Morris T.A., Sood N. (2016). Antithrombotic Therapy for VTE Disease: CHEST Guideline and Expert Panel Report. Chest.

[5] Sutphin P.D., Reis S.P., McKune A., Ravanzo M., Kalva S.P., Pillai A.K. (2015). Improving inferior vena cava filter retrieval rates with the define, measure, analyze, improve, control methodology. J. Vasc. Interv. Radiol..

[6] Sarosiek S., Crowther M., Sloan J.M. (2013). Indications, complications, and management of inferior vena cava filters: The experience at an academic medical center. Am. J. Med..

[7] Geisbüsch P., Benenati J.F., Peña C.S., Couvillon J., Powell A., Gandhi R., Samuels S., Uthoff H. (2012). Retrievable IVC filters: Factors that affect retrieval success. Cardiovasc. Intervent. Radiol..

[8] Kaufman J.A., Kinney T.B., Streiff M.B., Sing R.F., Proctor M.C., Becker D., Cippole M., Comerota A.J., Millward S.F., Rogers F.B. (2012). Guidelines for the use of retrievable and permanent inferior vena cava filters: Report from the Society of Interventional Radiology multidisciplinary consensus conference. J. Vasc. Interv. Radiol..

[9] Watson J.D.B., Morrison J.J., McNamee I.L., Evans T.W., Rasmussen T.E. (2013). Impact of a dedicated inferior vena cava (IVC) filter clinic on IVC filter retrieval rates. J. Vasc. Surg..

[10] Minocha J., Idakoji I., Riaz A., Karp J., Gupta R., Chrisman H.B., Salem R., Ryu R.K., Lewandowski R.J. (2010). Improving inferior vena cava filter retrieval rates with the use of a dedicated inferior vena cava filter registry. J. Vasc. Interv. Radiol..

[11] PREPIC Study Group (2005). Eight-year follow-up of patients with permanent vena cava filters in the prevention of pulmonary embolism: The PREPIC (Prévention du Risque d’Embolie Pulmonaire par Interruption Cave) randomized study. Circulation.

[12] Caplin D.M., Nikolic B., Kalva S.P., Ganguli S., Saad W.E.A., Zuckerman D.A., Society of Interventional Radiology Standards of Practice Committee (2011). Quality improvement guidelines for the performance of inferior vena cava filter placement for the prevention of pulmonary embolism. J. Vasc. Interv. Radiol..

[13] Kuo W.T., Robertson S.W., Odegaard J.I., Hofmann L.V. (2013). Complex retrieval of fractured, embedded, and penetrating inferior vena cava filters: Techniques for removal and clinical outcomes. J. Vasc. Interv. Radiol..

[14] Mismetti P., Laporte S., Pellerin O., Ennezat P.-Y., Couturaud F., Elias A., Falvo N., Meneveau N., Quéré I., Roy P.-M. (2015). Effect of a Retrievable Inferior Vena Cava Filter Plus Anticoagulation vs Anticoagulation Alone on Risk of Recurrent Pulmonary Embolism: The PREPIC2 Randomized Clinical Trial. JAMA.

